# Automatic attraction of visual attention by supraletter features of former target strings

**DOI:** 10.3389/fpsyg.2014.01383

**Published:** 2014-11-27

**Authors:** Søren Kyllingsbæk, Sven Van Lommel, Thomas A. Sørensen, Claus Bundesen

**Affiliations:** ^1^Department of Psychology, Center for Visual Cognition, University of CopenhagenCopenhagen, Denmark; ^2^Katholieke Universiteit Leuven – University of LeuvenLeuven, Belgium; ^3^Department of Communication and Psychology, Aalborg UniversityAalborg, Denmark

**Keywords:** attention, visual search, capture, visual perception, letters

## Abstract

Observers were trained to search for a particular horizontal string of three capital letters presented among similar strings consisting of exactly the same letters in different orders. The training was followed by a test in which the observers searched for a new target that was identical to one of the former distractors. The new distractor set consisted of the remaining former distractors plus the former target. On each trial, three letter strings were displayed, which included the target string with a probability of 0.5. In Experiment 1, the strings were centered at different locations on the circumference of an imaginary circle around the fixation point. The training phase of Experiment 2 was similar, but in the test phase of the experiment, the strings were located in a vertical array centered on fixation, and in target-present arrays, the target always appeared at fixation. In both experiments, performance (*d’*) degraded on trials in which former targets were present, suggesting that the former targets automatically drew processing resources away from the current targets. Apparently, the two experiments showed automatic attraction of visual attention by supraletter features of former target strings.

## INTRODUCTION

Following the lead of Shiffrin and Schneider (1977, Experiment 4d), Kyllingsbæk et al. (2001) explored the extent to which visual features of alphanumeric characters gain in pertinence (propensity to attract attention to characters with the given features) by prolonged and consistent training in visual search for characters with these features. In a simple and instructive experiment (Kyllingsbæk et al., 2001, Experiment 4), six different (types of) letters (H, N, L, T, X, and Z) were used as stimuli. For each participant, one of the six different letters served as the target throughout the training phase, while the other five letters served as distractors. On each trial, a circular array of letters was presented briefly, followed by a pattern mask. The participant’s task was to indicate whether the target letter appeared in the array. No time pressure was imposed on the response. Training sessions were run during four successive days. On the fifth day of the experiment, the target and distractor sets were redefined. One of the five letters that had been used as distractors during the training was selected to be the new target. The new distractor set consisted of the four remaining former distractors plus the former target. The presentation of the former target, instead of a former distractor, caused a decrement in *d’* averaging 0.15 units (*breakthrough* effect). Apparently, the former target letter automatically drew processing resources away from the current target.

The results of Schneider and Shiffrin (1977) and Kyllingsbæk et al. (2001) suggest that visual attention can be attracted by shapes as complex as those of individual alphanumeric characters. As noted by Kyllingsbæk et al. (2001), other evidence seems to suggest that the initial allocation of attention to items in a visual display is insensitive to words of four letters or more. Bundesen et al. (1997) presented observers with briefly exposed visual displays of words, which were common first names with a length of four to six letters. In the primary experiment, each display consisted of four words: two names shown in red and two shown in white. The observer’s task was to report the red names (targets), but ignore the white ones (distractors). On some trials the observer’s own name appeared as a display item (target or distractor). Presentation of the observer’s name as a distractor caused no more interference with report of targets than did presentation of other names as distractors. Apparently, visual attention was not automatically attracted by the observer’s own name. By contrast, a supplementary single-stimulus identification experiment showed that observers were more accurate in reading their own name than in reading other names (for a similar finding, see Shapiro et al., 1997).

If a visual 4-letter word could attract attention automatically, we would expect the attention of an observer with a 4-letter name to be attracted automatically by his or her own name (see Moray, 1959). As suggested by Bundesen et al. (1997), the contrast between findings with single letters and digits and findings with short words may be explained by assuming that visual attention can be attracted by individual alphanumeric characters, but not by shapes as complex as those of 4-letter words. To further explore this issue, we conducted two new experiments investigating attentional effects of prolonged search for strings of three letters. We chose 3-letter strings rather than 4-letter strings to decrease the complexity of the stimuli and thus increase the likelihood that they would be able to attract attention after training. The letter strings all contained the same three letters and could only be distinguished from each other by considering the ordering of the three letters comprising each string (supraletter features).

## EXPERIMENT 1

In Experiment 1, the participants trained for 2 days searching for a pre-designated 3-letter target-string presented among similar strings consisting of the same letters in different orders. On the third day the task was changed to one of searching for one of the former distractors while ignoring the former target string.

The stimulus material was designed so that it was impossible to discriminate any of the stimulus strings from all of the remaining ones by considering only simple features of individual letters or identities of the individual letters making up the strings. The stimuli were defined as all the possible ordered combinations of the letters *E*, *L*, and *O*, which yielded six 3-letter strings: *ELO, EOL, LEO, LOE, OEL,* and *OLE*. The only way in which any of the six strings could be discriminated from the rest of the stimuli was by considering the ordering of the three letters comprising the string.

Three of the stimuli were common Danish first names (*ELO, LEO,* and *OLE*), whereas the rest of the stimuli were non-words in Danish. Propensity to attract attention may develop more easily for familiar stimuli such as letters or words (see Czerwinski et al., 1992) than for less familiar stimuli such as non-words. Our stimulus material made it easy to test this possibility.

### METHOD

#### Participants

Five students (all females) from the University of Copenhagen participated in the experiment. Each participant was paid DKK 100 ($14) per hour. The ages of the participants ranged between 18 and 25 years. All participants had normal or corrected-to-normal visual acuity. The experiment was approved by the local ethical committee of the Department of Psychology, University of Copenhagen.

#### Stimuli

Six letters strings (*ELO, EOL, LEO, LOE, OEL,* and *OLE*) were used as stimulus material. Each stimulus frame contained eight possible stimulus positions (N, NE, E, SE, S, SW, W, NW) on the circumference of an imaginary circle centered on fixation. Each stimulus display contained three stimuli, which were distributed randomly across the eight positions. The distance from the center of a letter string to a small white fixation cross at the center of the screen was 40 mm (1.9°). The width and height of the letter strings were 18 (0.9°) and 8 mm (0.4°), respectively. All stimuli were presented in white on a black background at a viewing distance of 1.2 m.

#### General procedure

**FIGURE 1:**
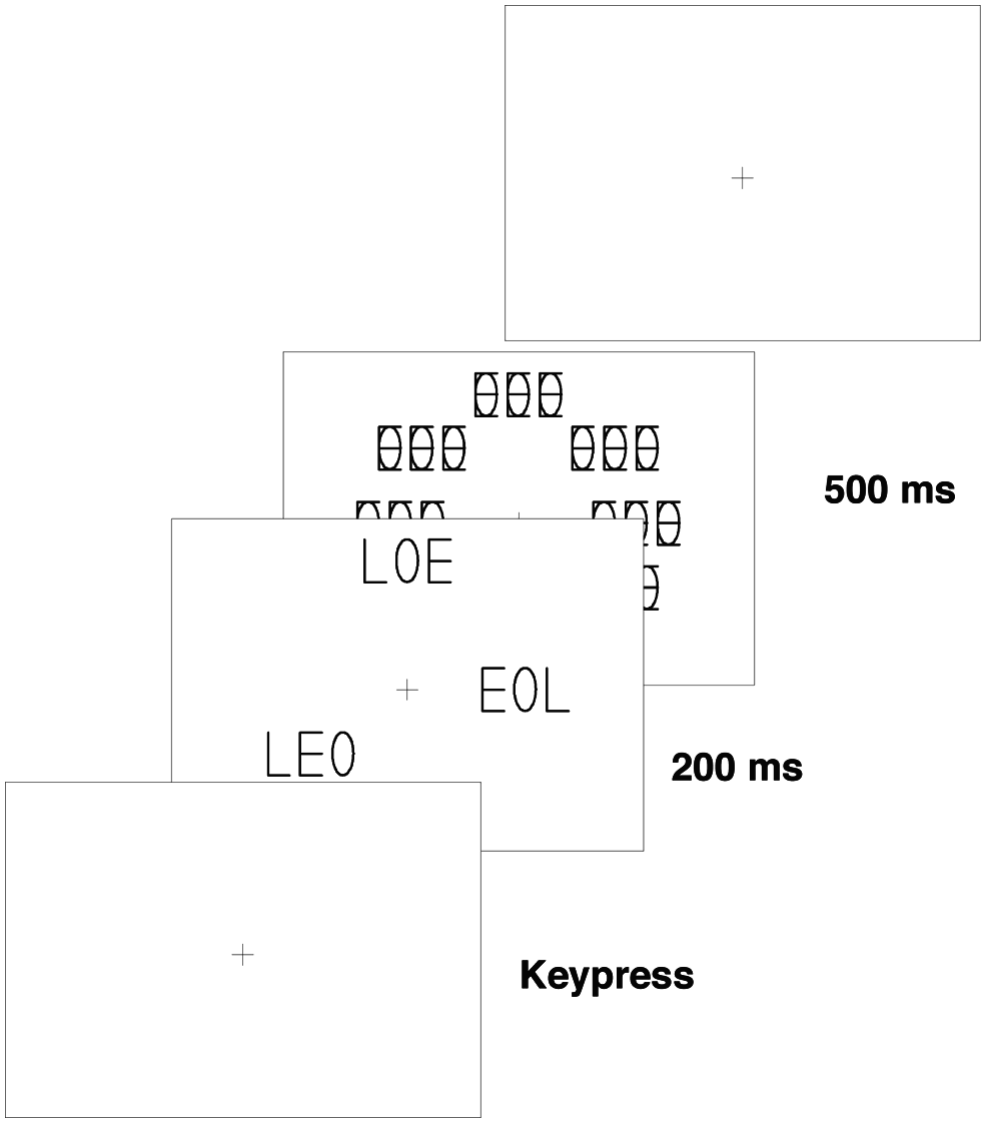
**Procedure used in the training and test phase of Experiment 1 and in the training phase of Experiment 2**.

The experiments were run on a CRT controlled by a PC. The participants were seated in a semi-darkened room 1.2 m from the screen. The participant started each trial by first fixating the fixation cross and when ready pressing a key, which immediately released a brief 200-ms exposure of the stimulus frame. The stimulus frame was immediately succeeded by a 500-ms exposure of a frame with eight masks, one at each of the eight possible stimulus positions (see **Figure 1**). The participant’s task was to indicate whether a pre-designated target was present in the stimulus frame. Participants responded *present* by pressing the right key and *absent* by pressing the left key of a response box. A short warning sound was given as feedback when an error was made.

#### Training

For each participant, one of the six strings served as the target throughout the training phase, while the other five letter strings served as distractors. On each trial, the target appeared in the display with a probability of 0.5.

One session consisted of 2,000 trials (100 blocks of 20 trials each) and took about 2 h. For each trial, three strings were presented and the distractors were randomly drawn without replacement from the set of five distractor strings. Two training sessions were run during two successive days.

#### Test

On the third day of the experiment, target and distractor sets were redefined. One of the five strings that had been used as distractors during the training was selected to be the new target. The new distractor set consisted of the four remaining former distractors plus the former target string. One test session was run with the new target and distractor sets. The former target appeared (once per display) in one half of the stimulus displays. Except as noted the procedure during the test phase was the same as during the training. Thus, the probability that the former target appeared in a stimulus display was exactly the same as the probability that any other particular member of the new distractor set appeared in the display.

### RESULTS

**FIGURE 2:**
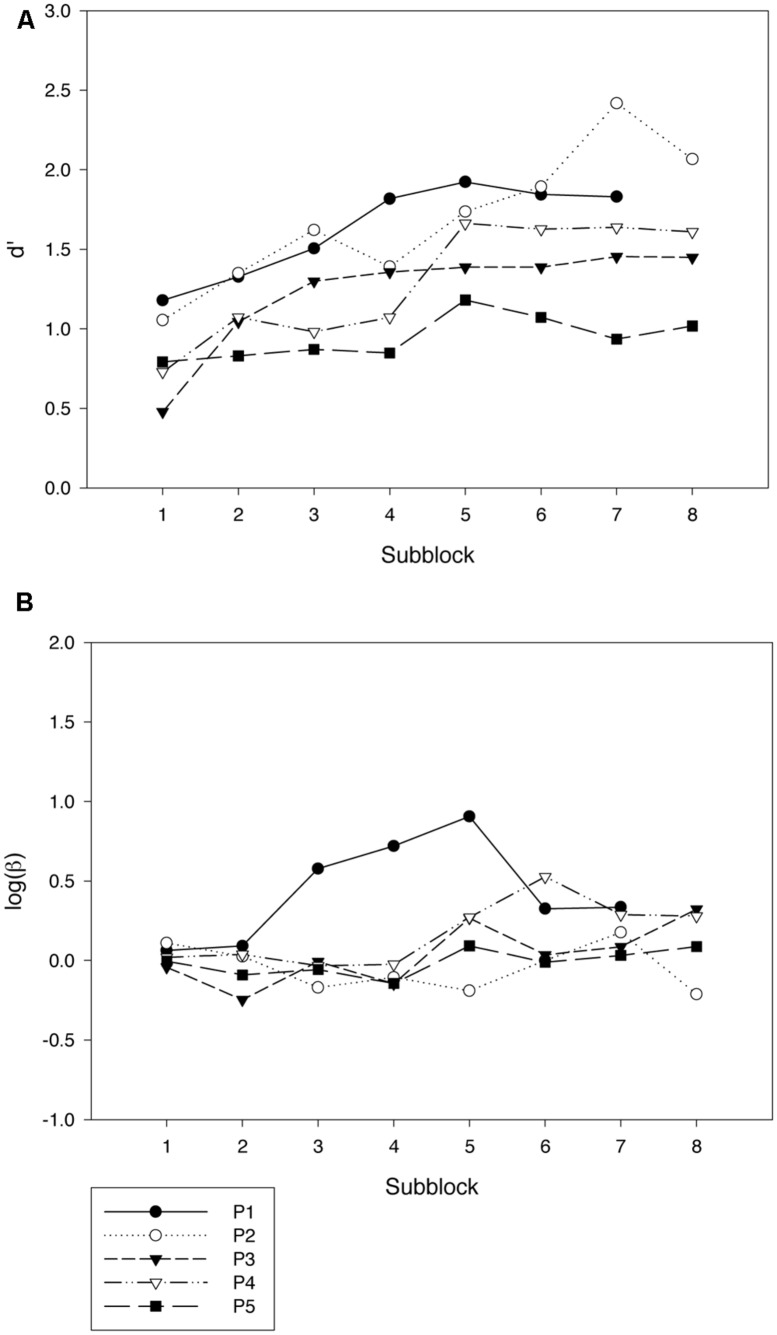
**Results from the training phase of Experiment 1.** Each graph depicts the data for one participant by subblocks of 500 trials. **(A)** shows variations in sensitivity (*d’*), and **(B)** shows variations in bias (logβ). As can be seen from the graphs for Participant 1 (P1), the data from the last subblock was lost for this participant.

The error rates were analyzed by use of *signal-detection theory* (Green and Swets, 1966) to disentangle variations in sensitivity (measured by parameter *d’*) from variations in response bias (measured by the natural logarithm of parameter β). Learning curves for each participant are shown with respect to both sensitivity (**Figure 2**, Panel A) and bias (**Figure 2**, Panel B). The data were split into subblocks of 500 trials each and the following analyses were also done with this division of the data. A linear regression analysis across the five participants showed a significant increase in sensitivity during the training period [*F*(1,3) = 16.72, *p* < 0.05]^1^. The rate of increase in *d’* averaged 0.11 units per subblock. The linear trend in log β as a function of number of session did not reach significance [*F*(1,3) = 3.21, *p* = 0.17].

**FIGURE 3:**
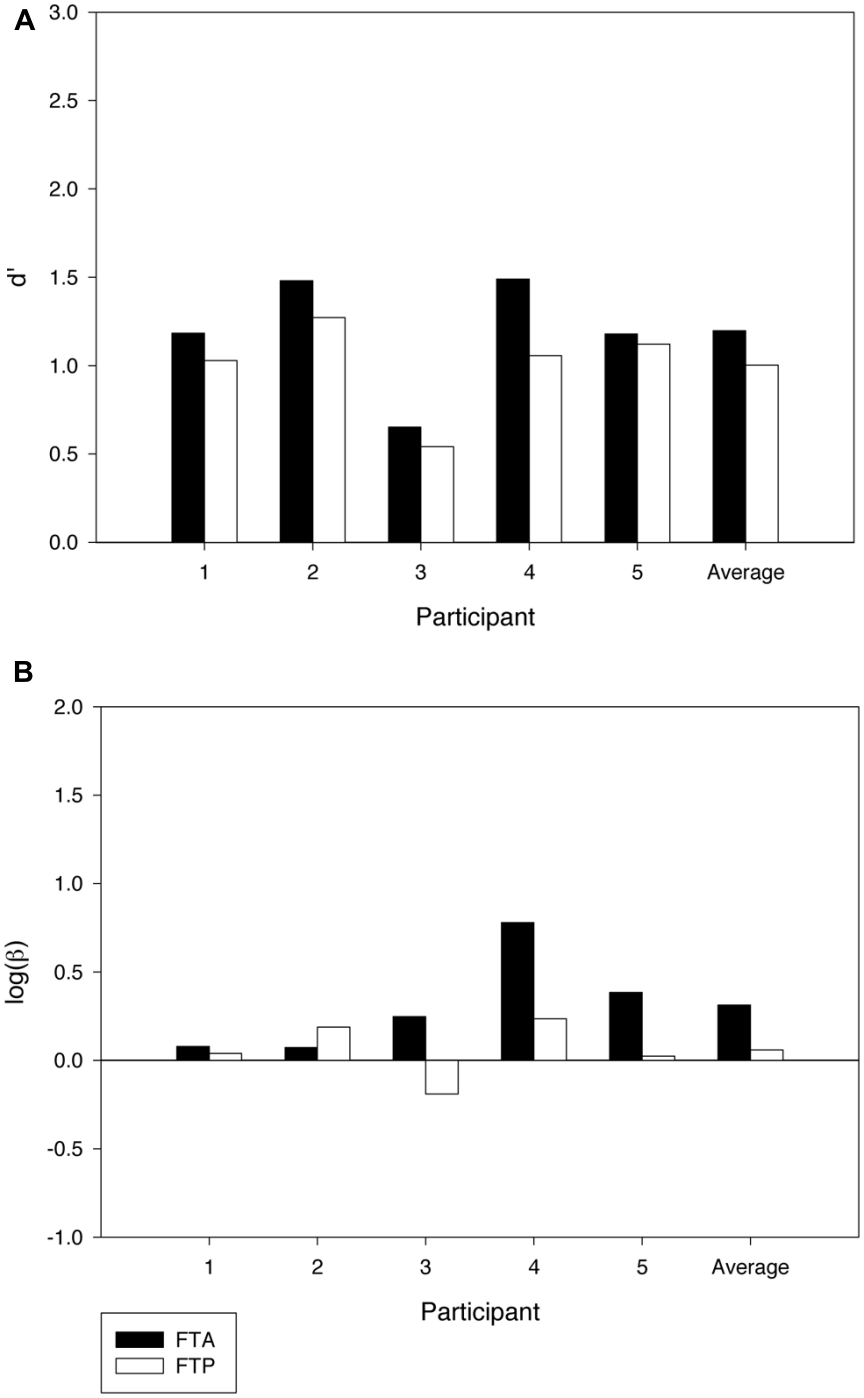
**Results from the test phase of Experiment 2.** The data are separately shown for trials in which the former target was absent (FTA) and present (FTP), respectively. **(A)** shows variations in sensitivity (*d’*) across participants, and **(B)** shows variations in bias (logβ).

The effects of former targets on sensitivity and bias in the test phase are illustrated in **Figure 3**. We computed sensitivity and bias values for the two conditions by first separating trials where the former target was present and absent, respectively. We then computed hits and false alarm rates within the two sets of trials and from these sensitivity and bias values for the two conditions. As can be seen in Panel A of **Figure 3**, sensitivity was lower when the former target was present than when the former target was absent. The effect of the former target was significant [*t*(4) = 2.97, *p* < 0.05] and present in all the five participants. The decrement in *d’* averaged 0.19 units, range 0.06–0.43. The effect on bias bordered on significance [*t*(4) = 2.03, *p* = 0.06] suggesting that participants may have been more conservative in the training phase compared to the test phase (see **Figure 3B**). When testing if the effect on *d’* depended on whether the former target was a word or a non-word, we found no significant difference [*t*(3) = 1.12, *p* = 0.35] (see also **Table 1**). Of course, with only five participants, the null result may be a Type II error due to lack of power (but see Experiment 2).

**Table 1:** Targets during training and test for participants in Experiments 1 and 2.

Participant	Trainingtarget	Testtarget	Is training target a word?	Decrementin *d’* (FTA – FTP)
**Experiment 1**				
1	LEO	ELO	+	0.16
2	LOE	OLE		0.21
3	ELO	LEO	+	0.11
4	EOL	OEL		0.43
5	LEO	ELO	+	0.06
**Experiment 2**				
1	ELO	LOE	+	0.04
2	EOL	LEO		0.07
3	LOE	OEL		0.03
4	OEL	ELO		-0.05
5	OLE	EOL	+	0.01
6	ELO	OEL	+	0.09
7	EOL	OLE		0.05
8	LEO	EOL	+	0.01

### DISCUSSION

The decrement in sensitivity observed when the former target was presented as a distractor extended the findings reported by Shiffrin and Schneider (1977) and Kyllingsbæk et al. (2001). The magnitude of the decrement we found in *d’* (0.19 units) was comparable in magnitude to the decrement (0.25 units) found by Kyllingsbæk et al. (2001, Experiment 4) in a study of search for a single letter target in displays of three letters. We found no evidence for differential effects of presentation of former targets depending on whether these were words or non-words.

The main finding from Experiment 1 was that the breakthrough of former targets demonstrated by Shiffrin and Schneider (1977) and Kyllingsbæk et al. (2001) for individual alphanumeric characters could be obtained for former targets that were 3-letter strings defined neither by visual features of individual letters, nor by the global shapes of individual letters, but, apparently, by features that reflected the ordering of the letters in the target string: supraletter visual features.

## EXPERIMENT 2

The results of Experiment 1 might be interpreted not as a result of automatic attraction of attention by the former 3-letter target, but rather as attention getting stuck at the former target when accidentally encountered, assuming, for example, that attention is allocated to the display items in a random order. To test this hypothesis, we fixed the location of the target in the test phase of Experiment 2.

The training phase of Experiment 2 was identical to the one used in Experiment 1. However, in the test phase the display setup was changed so that the target could be selected by location. Instead of a circular search display with varying stimulus locations, the three display elements were always located in a vertical column centered at fixation. Further, the new target string always appeared at the central location if present (known by the participants), whereas the former target never appeared at the central location (not known by the participants). If participants were able to ignore the former target by attending exclusively to the string presented at the central location, we should expect no effect of presentations of the former target.

### METHOD

#### Participants

Eight students (four females and four males) from the University of Copenhagen participated in the experiment. Each participant was paid DKK 100 ($14) per hour. The ages of the participants ranged between 17 and 29 years. All participants had normal or corrected-to-normal visual acuity. The experiment was approved by the local ethical committee of the Department of Psychology, University of Copenhagen.

#### Stimuli

**FIGURE 4:**
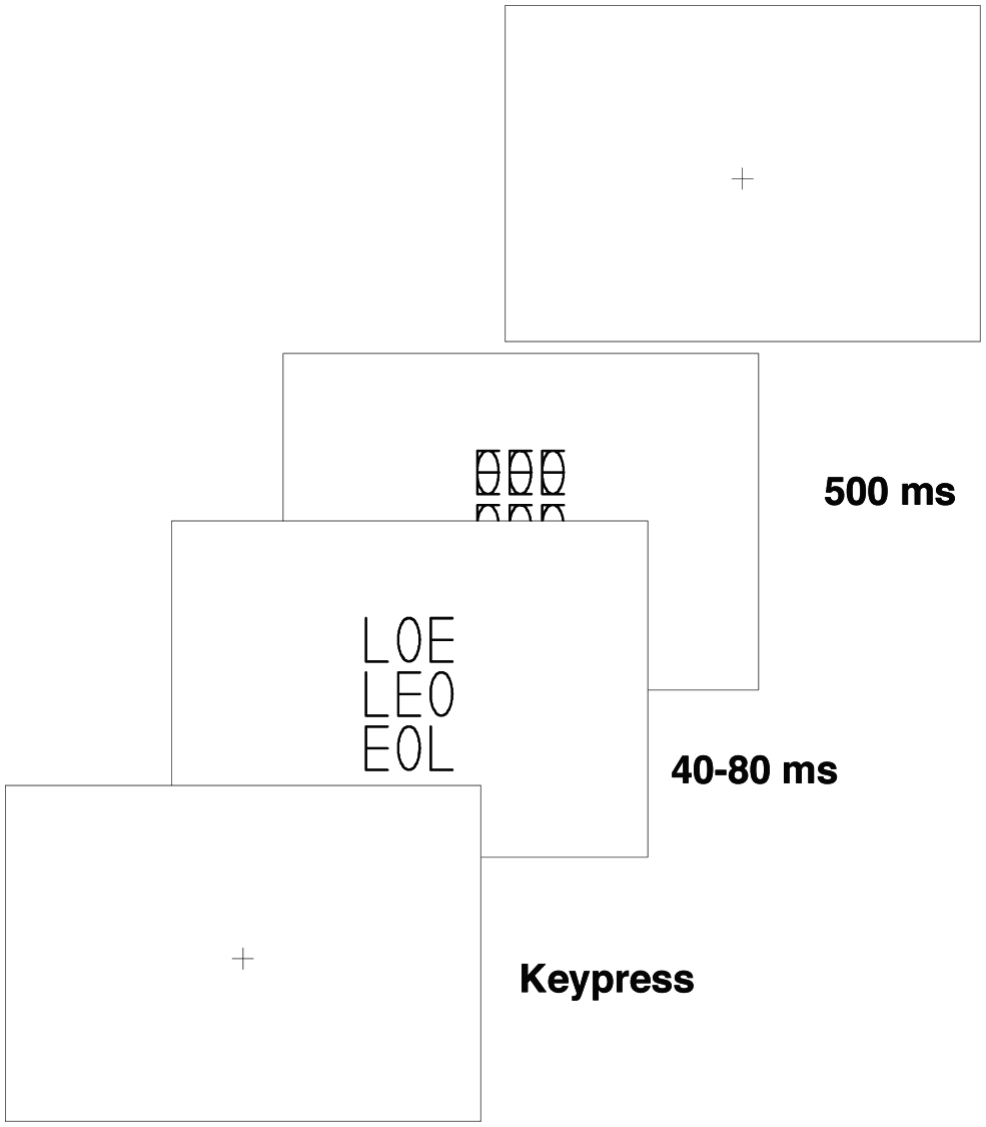
**Procedure used in test phase of Experiment 2 (see **Figure 1** for the procedure for the training phase of Experiment 2)**.

The stimulus material was the same as the one used in Experiment 1. Only the stimulus frame during the test phase was different. In the test phase of Experiment 2, the three letter strings were positioned in a vertical column centered at fixation (see **Figure 4**). The center-to-center distance between the strings was 12 mm (0.6°).

#### Procedure

The procedure during the training phase was identical to that of Experiment 1. In the test phase, however, the participants were instructed to attend exclusively to the middle one of the three strings presented (i.e., the string presented at fixation). Participants were told that the new target string would always appear at the central location if present in the display. Participants thus had a clear incentive to attend to the stimulus at the central location and ignore the two flanking distractor strings. Further, the former target string never appeared at the central location. Participants were not made aware of this fact and none reported having noticed it when questioned after the end of the experiment. The exposure duration was calibrated before the start of the test phase for each participant to prevent ceiling and floor effects. The exposure duration ranged between 40 and 80 ms across the eight participants.

#### Design

Again one of the six letter strings was designated as target for each participant and two blocks of 2,000 trials were run as training. The test phase comprised 2,000 trials similarly to the test phase in Experiment 1.

Because of the constraint that the former target could not appear at the central location during the test phase, the new target had to be selected from a particular subset of the five distractor strings from the training phase in order to prevent participants from using a strategy whereby the new target string could be identified by looking for only one of the letters in the string. For example, if the former target was *OLE*, the new target was either *EOL* or *LEO*. That is, the new target was one of the two strings in which neither *O* appeared as the first letter (i.e., as in *OEL*), *L* appeared as second letter (i.e., as in *ELO*), or *E* appeared as the third letter (i.e., as in *LOE*). If any of the strings *OEL*, *ELO*, or *LOE* had been chosen as new target, participants would have been able to identify the new target at the central location by looking for an *O* at the first position in the string, an *L* at the second, or an *E* at the third, respectively.

### RESULTS

**FIGURE 5:**
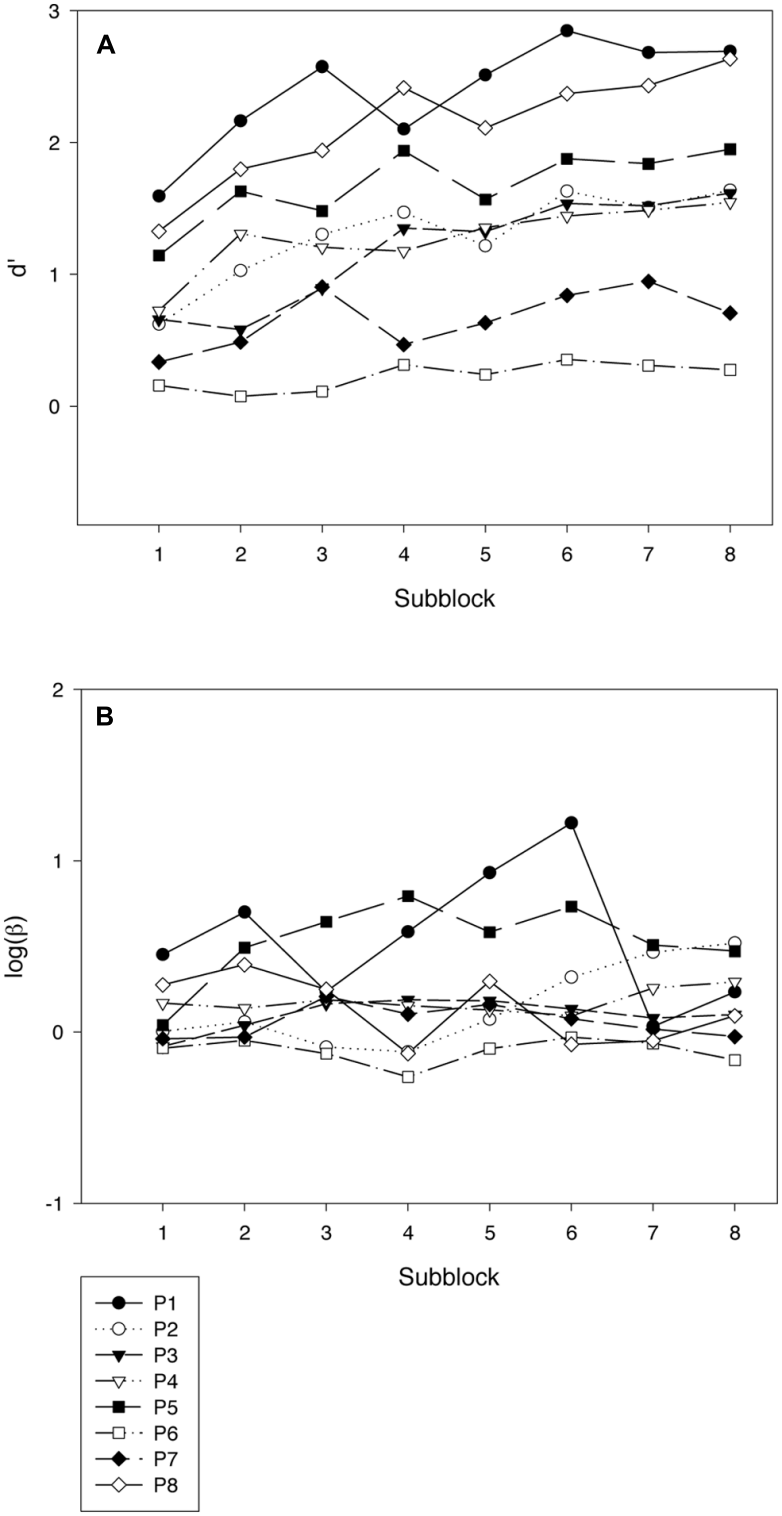
**Results from the training phase of Experiment 2.** Each graph depicts the data for one participant by subblocks of 500 trials. **(A)** shows variations in sensitivity (*d’*), and **(B)** shows variations in bias (logβ).

**Figure 5** shows learning curves for sensitivity (Panel A) and bias (Panel B). As in Experiment 1, there was a strong and significant linear trend for sensitivity [*F*(1,7) = 41.36, *p* < 0.001], but no significant trend for bias (*F* < 1). The average rate of increase in *d’* was 0.11 units per subblock of 500 trials.

**FIGURE 6:**
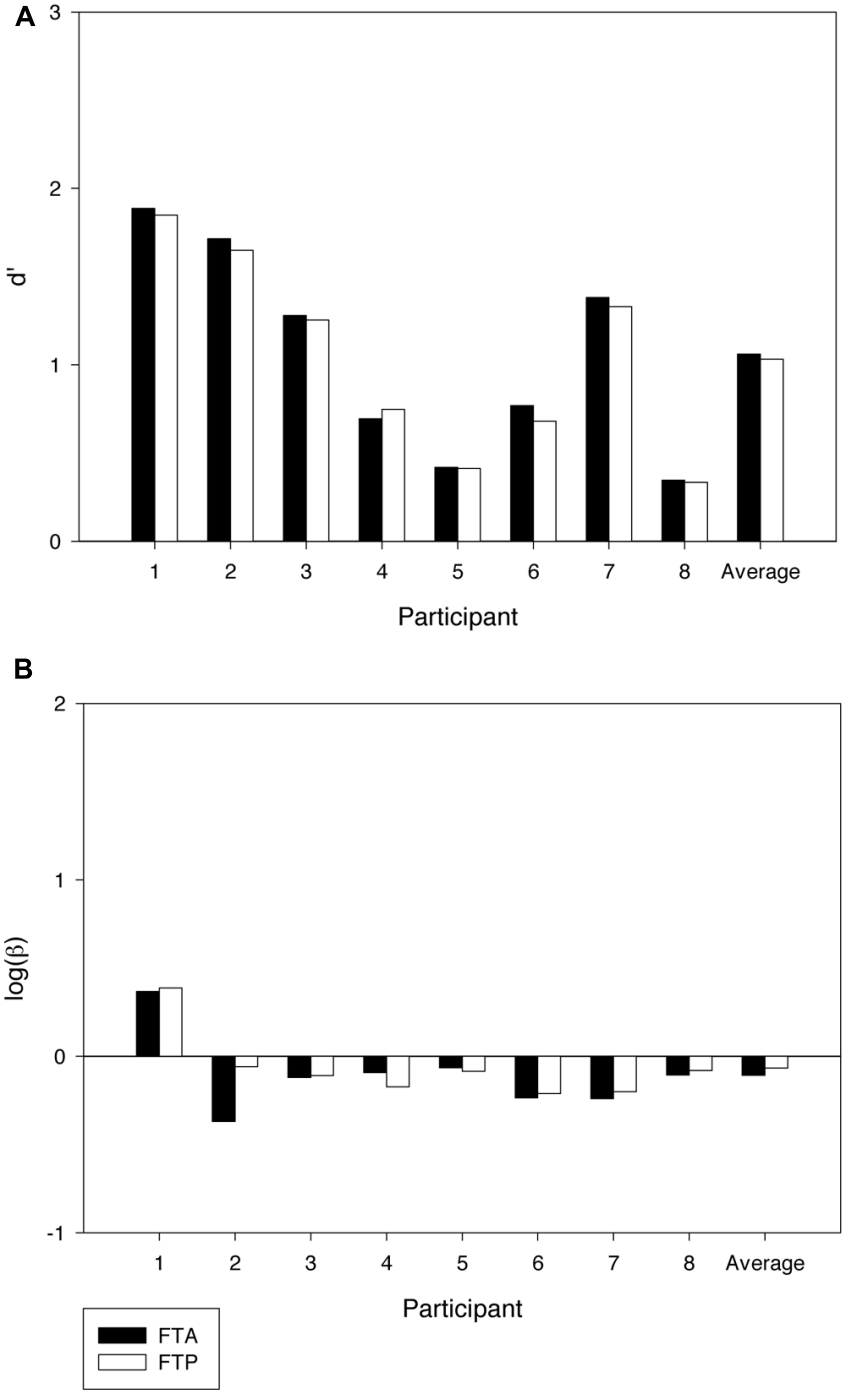
**Results from the test phase of Experiment 2.** The data are separately shown for trials in which the former target was absent (FTA) and present (FTP), respectively. **(A)** shows variations in sensitivity (*d’*) across participants, and **(B)** shows variations in bias (logβ).

The effect of former targets on sensitivity and bias in the test phase is shown in **Figure 6**. Panel A shows that *d’* was again lower when the former target was present compared to trials in which it was absent [*t*(7) = 1.94, *p* < 0.05]. The effect was observed in seven out of the eight participants. The decrement in *d’* averaged 0.03 units. The effect on bias did not reach significance [*t*(7) = -1.01, *p* = 0.35]. Again, we found no effect on *d’* of whether the former target was a word or a non-word [*t*(6) = -0.423, *p* = 0.69] (see also **Table 1**).

### DISCUSSION

In Experiment 2, uncertainty concerning the possible target location was reduced to a minimum by using a fixed location centered at fixation. The former target never appeared at this location, but only at the two flanking locations. As in Experiment 1, presentation of the former target impeded detection of a simultaneously presented current target. The decrement in *d’* found in Experiment 2 (0.03 units) was smaller than the decrement found in Experiment 1 (0.19 units), but still statistically significant.

## GENERAL DISCUSSION

Experiments 1 and 2 provided clear evidence of automatic attraction of visual attention by supraletter features of letter strings following prolonged and consistent practice in search for these targets. Either experiment replicated the breakthrough effect of Shiffrin and Schneider (1977, Experiment 4d) and Kyllingsbæk et al. (2001) with a stimulus ensemble consisting of 3-letter strings that were constructed in such a way that it was impossible to determine whether a string was a target or a distractor by testing for either features of individual letters or presence of particular individual letters within the string. Thus, because the former targets and other distractors consisted of exactly the same letters, our findings suggest that supraletter visual features that reflected the ordering of the letters in the targets gained pertinence (propensity to attract attention to objects with the given features) during the training (see, e.g., Bundesen, 1990; Nordfang et al., 2013).

The nature and complexity of the supraletter visual features in question is still a matter of speculation. Most obviously, having the shape of the 3-letter target string (e.g., ELO) as a whole may be one supraletter visual feature that gained pertinence and, accordingly, enhanced the attentional weight of the target during training. However, supraletter visual features need not be complex. Containing a particular bigram (ordered pair of letters such as EL or LO) within the target string, or containing a bigram with particular features, is a more simple supraletter visual feature that also may have gained pertinence and, thereby, enhanced the attentional weight of the target during training (see Dehaene et al., 2005, for a proposal for a neural code for written words in which bigrams, including “open bigrams,” have a pivotal role). Indeed, the supraletter visual features that gained pertinence could in principle have been any features of multiletter units that were useful in discriminating the target string of letters from the distractor strings.

The results found by Shiffrin and Schneider (1977) have had a strong impact on the development of general theories of attention (see, e.g., Duncan, 1980; Treisman, 1988; Duncan and Humphreys, 1989; Bundesen, 1990; van der Heijden, 1992; Wolfe, 1994; Lavie, 1995; Schneider, 1995, 1999). Proponents of late selection theories of attention (e.g., Shiffrin and Schneider, 1977) have argued that if a particular type of stimuli automatically attracts attention, recognition of this type of stimuli must be possible preattentively and in parallel across all objects in the visual field (see also Kyllingsbæk and Bundesen, 2007). A weaker and safer claim is that if a particular type of stimuli automatically attracts attention, retrieval of evidence that stimuli belong to the type in question must be possible preattentively and in parallel across the visual field. Thus, the results of the present experiments suggest that simultaneously presented visual stimuli defined by supraletter features can be compared in parallel against representations in visual long-term memory.

## Conflict of Interest Statement

The authors declare that the research was conducted in the absence of any commercial or financial relationships that could be construed as a potential conflict of interest.

## Footnotes

1 The analyses of the training sessions were based on only the data from Participants 2–5, because the data from the last subblock was lost for Participant 1.
